# A microfluidic platform enabling single-cell RNA-seq of multigenerational lineages

**DOI:** 10.1038/ncomms10220

**Published:** 2016-01-06

**Authors:** Robert J. Kimmerling, Gregory Lee Szeto, Jennifer W. Li, Alex S. Genshaft, Samuel W. Kazer, Kristofor R. Payer, Jacob de Riba Borrajo, Paul C. Blainey, Darrell J. Irvine, Alex K. Shalek, Scott R. Manalis

**Affiliations:** 1 Koch Institute for Integrative Cancer Research, Massachusetts Institute of Technology, Cambridge, Massachussets 02139, USA; 2 Department of Biological Engineering, Massachusetts Institute of Technology, Cambridge, Massachussets 02139, USA; 3 Department of Materials Science and Engineering, Massachusetts Institute of Technology, Cambridge, Massachussets 02139, USA; 4 Ragon Institute of Massachusetts General Hospital, Massachusetts Institute of Technology, and Harvard, Cambridge, Massachussets 02139, USA; 5 Department of Chemistry, Massachusetts Institute of Technology, Cambridge, Massachussets 02139, USA; 6 Institute for Medical Engineering & Science, Massachusetts Institute of Technology, Cambridge, Massachussets 02139, USA; 7 Broad Institute of MIT and Harvard, Cambridge, Massachussets 02142, USA; 8 Microsystems Technology Laboratory, Massachusetts Institute of Technology, Cambridge, Massachussets 02139, USA; 9 Howard Hughes Medical Institute, Chevy Chase, Maryland 20815, USA; 10 Harvard-MIT Division of Health Sciences and Technology, Massachusetts Institute of Technology, Cambridge, Massachussets 02139, USA; 11 Department of Immunology, Massachusetts General Hospital, Boston, Massachussets 02114, USA; 12 Department of Mechanical Engineering, Massachusetts Institute of Technology, Cambridge, Massachussets 02139, USA

## Abstract

We introduce a microfluidic platform that enables off-chip single-cell RNA-seq after multi-generational lineage tracking under controlled culture conditions. We use this platform to generate whole-transcriptome profiles of primary, activated murine CD8+ T-cell and lymphocytic leukemia cell line lineages. Here we report that both cell types have greater intra- than inter-lineage transcriptional similarity. For CD8+ T-cells, genes with functional annotation relating to lymphocyte differentiation and function—including Granzyme B—are enriched among the genes that demonstrate greater intra-lineage expression level similarity. Analysis of gene expression covariance with matched measurements of time since division reveals cell type-specific transcriptional signatures that correspond with cell cycle progression. We believe that the ability to directly measure the effects of lineage and cell cycle-dependent transcriptional profiles of single cells will be broadly useful to fields where heterogeneous populations of cells display distinct clonal trajectories, including immunology, cancer, and developmental biology.

## Abstract (web summary)

Existing single-cell RNA-seq methods provide the transcriptome of a cellular phenotype at a single time point. Here, Kimmerling *et al*. present a microfluidic platform that enables off-chip single-cell RNA-seq after multigenerational lineage tracking under controlled culture conditions.

## Graphical abstract

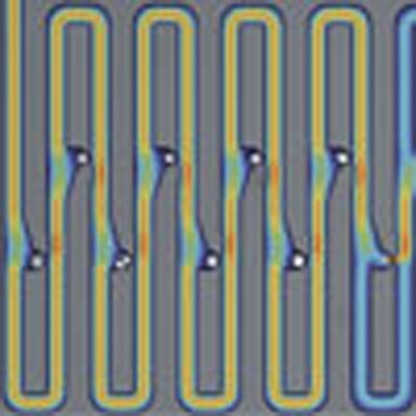

The development of single-cell RNA-seq has led to a new degree of resolution in the characterization of complex, heterogeneous biological systems^1^. Complimentary technical advances in single-cell isolation using micromanipulation, microfluidics and fluorescence activated cell sorting have further enabled the coupling of traditional measurements of cellular phenotype, such as immunofluorescence staining and optical microscopy, with transcriptional profiles^2^. Together, these approaches have provided crucial insights into the transcriptional heterogeneity of cancer^3^, immune^4^ and pluripotent stem cells^5^.

Because these single-cell isolation platforms rely on single time point measurements, they provide only an instantaneous snapshot of cellular phenotype to link to a transcriptional signature. In addition to understanding the transcriptional heterogeneity within a population of cells, the mechanisms for generating this heterogeneity over time are also of critical importance. For instance, a cornerstone of adaptive immunity is the ability of single T-lymphocytes to generate diverse progeny that can both acutely respond to a specific antigen and provide long-term protection in the event of a future exposure. However, the mechanism by which this diversity is generated from a single founding cell remains a highly controversial topic^6^,^7^,^8^,^9^. Resolving the relative contributions of various models of T-cell differentiation—as well as generally defining the mechanisms by which a single cell gives rise to distinctly different progeny in various biological systems**—**requires a means of directly tracking single-cell lineage while making sensitive measurements of cell phenotype.

Recent developments in microfluidic technology have enabled new methods of capturing and culturing single cells^10^,^11^. When coupled with traditional imaging approaches, these systems offer a robust means of following cellular trajectories over time, but require experimental platforms that can reliably link these measurements to downstream single-cell gene expression profiles^12^,^13^. Alternatively, microfluidic devices which enable the efficient preparation of single-cell cDNA libraries for gene expression analysis—such as the Fluidigm C1 platform—currently lack the long-term culture, progeny capture and time-lapse imaging capabilities necessary to link these transcriptional measurements with lineage information.

Here, we present a microfluidic platform that allows direct association of these complementary data sets by enabling registered off-chip single-cell RNA-seq after multi-generational lineage tracking. We utilize this platform to collect single-cell transcriptional measurements for lineages of two well-studied model cell types: a mouse lymphocytic leukemia cell line (L1210) and primary murine CD8+ T-cells. These results reveal both lineage and cell cycle-dependent transcriptional signatures, and suggest that this platform may be broadly useful for studies of multigenerational development at the single cell level.

## Results

### Hydrodynamic trap array

**Figure 1:**
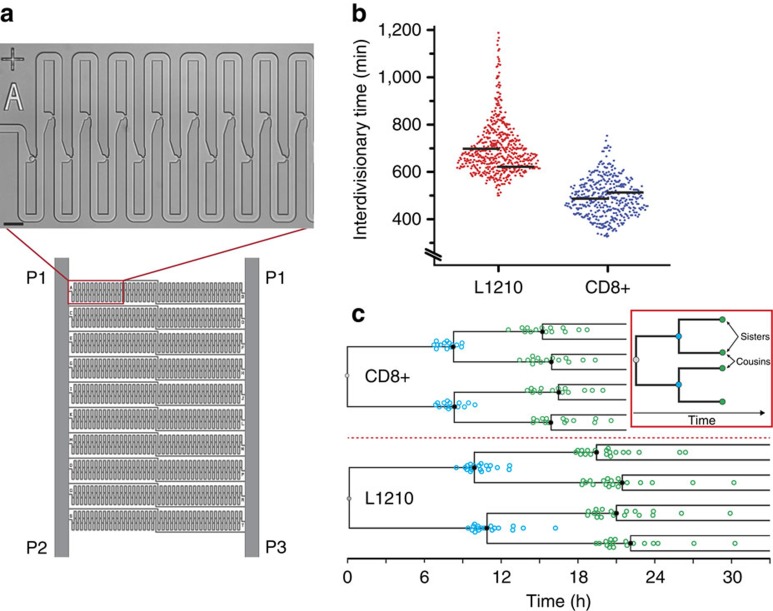
Microfluidic hydrodynamic trap array for single-cell lineage tracking. (**a**) Schematic representation of the hydrodynamic trap array consisting of 20 lanes of traps (inset shows an optical micrograph of a single trapped cell—scale bar, 20 μm). Independent control of upstream (P1) and downstream (P2, P3) pressures enables continuous perfusion for long-term proliferation (Supplementary Movie 2), as well as single cell release from the device (Supplementary Movie 3, Supplementary Note 1). (**b**) Single-cell interdivisionary time measurements for a murine leukemia cell line (L1210, *n*=526) and primary CD8+ T-cells isolated from C57BL/6J mice (CD8+, *n*=418) collected with the hydrodynamic trap array. Overlaid black bars indicate the mean interdivisionary time measured on-chip (left) and the doubling time measurements collected for bulk cultures (right) of each cell type (Supplementary Fig. 3b). (**c**) Overlay of lineage trees from single CD8+ T cells (*n*=15) and L1210 cells (*n*=20) established with time-lapse imaging in the hydrodynamic trap array. As a demonstration of lineage construction, one lineage for each cell type (black circles) has connecting lines indicating familial history. The first division observed in the device corresponds to time zero for each lineage with subsequent points corresponding to the second (blue circles) and third (green circles) divisions on-chip. The inset demonstrates how sister and cousin cell pairs are defined for cell lineages released for sequencing.

Our platform utilizes an array of hydrodynamic traps within a fluidic design optimized to capture and culture single cells for multiple generations on-chip (Fig. 1a). These trap structures rely on differences in hydrodynamic resistance between the trapping pocket and a bypassing serpentine channel to deterministically capture single cells (Supplementary Fig. 1a)^10^. To increase the throughput of the system, groups of traps are arranged as independently accessible lanes with bypass channels flanking either side. The application of independent upstream and downstream pressures (P1, P2 and P3) drives fluid flow through the device. By establishing unique pressure gradients along (P1–P2 and P1–P3) and across (P2–P3) the bypass channels, this fluidic design decouples the flow through the bypass channels from the flow across each lane of traps (Supplementary Fig. 2). As such, media can be rapidly and continuously perfused through the bypass channels while maintaining minimal flow across the traps in order to ensure constant nutrient repletion with low and uniform shear stress on the cells (Supplementary Fig. 1b). This independent flow control also allows for rapid buffer exchange without dislodging trapped cells, thus enabling on-chip implementation of standard cell staining techniques such as immunocytochemistry and fluorescent labelling.

For experimental operation, a single cell is loaded into each lane of the trap array (Methods, Supplementary Movie 1 and Supplementary Note 1). As the cell proliferates, its progeny are carried downstream and captured in subsequent unoccupied traps (Supplementary Movie 2). Time-lapse imaging of this process allows for the determination of single-cell proliferation kinetics and identification of lineal relationships between cells (Fig. 1b,c). Each lane of traps can accommodate up to 40 cells and thus enables lineage tracking for up to five generations (with an experimental duration determined by the doubling time of the cells under study). Our system is capable of sustaining cell growth over more than 72 h for both a murine lymphocytic leukemia cell line (L1210) and primary murine CD8+ T-cells, matching or surpassing time scales achievable with state-of-the-art methods for long-term culture of single suspension cells^12^,^14^. Furthermore, growth kinetics in the device are stable over multiple generations and consistent with doubling time measurements collected from bulk cultures, suggesting that the platform does not significantly perturb long-term cell growth (Fig. 1b, Supplementary Fig. 3).

### Single-cell release

In contrast to previous approaches, our fluidic design enables the retrieval of single cells after multiple generations of proliferation for downstream assays, thereby allowing single-cell transcriptomic data to be linked to lineage, proliferation kinetics and live-cell image data. Cells are retrieved by reversing the pressure differential across the trap lanes to collect cells in the bypass channel where they can be flushed out of the chip in <5 μl of buffer (Supplementary Movie 3, Supplementary Note 1). The parallel lanes of traps each have a slightly different applied pressure (P3) at which the flow direction changes and cells begin to exit (Supplementary Movie 4); this difference enables independent release of cells from each lane and increases the throughput of the system (Supplementary Note 1).

Using this retrieval method, single cells from lineages of L1210 and CD8+ T-cells were captured off-chip for single-cell RNA-seq^15^. Videos documenting this release process allow us to couple transcriptional profiles with lineal relationships and measurements of time since division for each cell. In contrast, previous approaches have relied on computational analysis of single-cell transcriptional profiles to construct putative lineal relationships^6^. This platform, however, enables direct observation of familial history upstream of single-cell RNA-seq and presents a direct means of determining lineage-dependent transcriptional characteristics.

### Lineage-dependent transcriptional profiles

**Figure 2:**
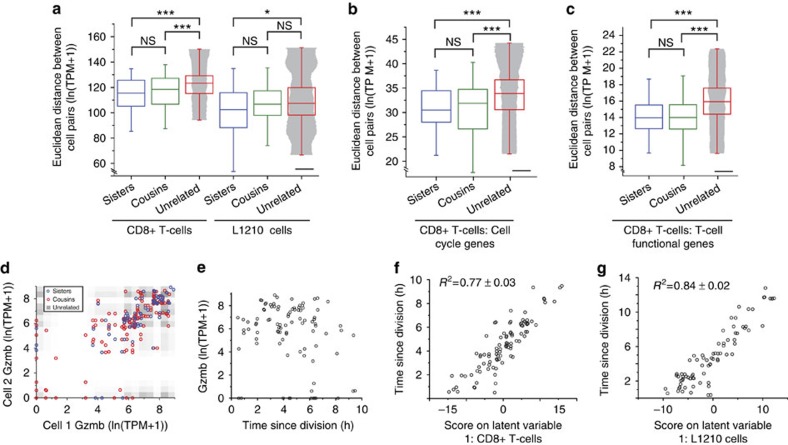
Single-cell RNA-seq with known lineage relationships and times since division. (**a**) Comparison of Euclidean distances (measured in log-transformed transcripts per million) between sister cells, cousin cells and unrelated cells for CD8+ T-cells (*n*=43, 73 and 4,544, respectively) and L1210 cells (*n*=37, 60 and 3,064, respectively) for the entire gene set (9,997 genes and 10,658 genes for CD8+ T-cells and L1210, respectively). Groups were compared with a Mann–Whitney *U*-test (Methods). The shaded area overlay on the unrelated cell pair measurements has a width corresponding to differences in time since division for these cells. The widths were constructed using a 250-point moving average of the pairwise differences in time since division for visual clarity. Scale bar, 3 h. (**b**) Same analysis as in (**a**) applied to a subset of genes with cell cycle-related gene annotations (688 genes total) for CD8+ T-cells. (**c**) Same analysis as in **a**,**b** applied to a subset of genes with gene annotations related to T-cell activation and function (142 genes total) for CD8+ T-cells. (Groups were compared with a Mann–Whitney *U*-test. After Bonferroni correction: *P<0.05, **P<0.01, ***P<0.001. Not-significant (NS) indicates a *P* value >0.05.) (**d**) Plot of Granzyme B expression levels (measured in log-transformed transcripts per million) in sister cell pairs (blue circles), cousin cell pairs (red circles) and unrelated cell pairs (grey scale density plot with darkest regions corresponding to highest relative occupancy). The correlation coefficients of related and unrelated cell pairs were compared with a Fisher’s *z* transformation (*P*=0.005). (**e**) Plot of Granzyme B expression levels as a function of the time since division for each single cell. (**f**,**g**) Plot of scores on the first latent variable of partial least squares regression models constructed with expression measurements as predictor variables and the time since division for each single cell as the response variable for CD8+ T-cells (**f**) and L1210 cells (**g**). The final models were constructed with genes corresponding to the top 300 VIP scores for each cell type (Supplementary Fig. 9, Methods).

Single cells were cultured for two generations on-chip before release for sequencing. This allowed us to define sister and cousin cell pairs for each lineage (Fig. 1c). To determine transcriptional patterns associated with lineage relationships in these cell systems, we compared gene expression similarity between related (that is, sisters and cousins) and unrelated cell pairs. Here, unrelated cell pairs refer to cells that were not derived from a common ancestor as observed in the device (that is, from different lineages). Transcriptional similarities were determined using Euclidean distance measurements to quantify the distance between two cells in log-transformed transcriptional space with smaller distances signifying more similar gene expression profiles (Methods). When comparing global expression levels in both cell types, sister cell pairs showed significantly higher transcriptional similarity than unrelated cell pairs (*P*=0.03 and *P*<0.001 for L1210 and CD8+ T-cells, respectively, Mann–Whitney *U*-test) (Fig. 2a). For CD8+ T-cells, this increased similarity also extended to cousin cell pairs (*P*<0.001, Mann–Whitney *U*-test). Since each founding CD8+ T-cell loaded in to the device represents a unique clone, these results suggest lower intra- than interclonal transcriptional variation for these cells (Fig. 2a–c). Conducting these analyses with Spearman distances, a rank-based metric of transcriptional similarity, yielded similar results across all comparisons (Supplementary Fig. 4). Interestingly, unsupervised clustering analysis of highly variable genes in both cell types successfully reconstructs lineage relationships for some, but not all, cells (Supplementary Fig. 5, Supplementary Note 2). This suggests that computational approaches alone may not offer the same efficacy in predicting single-cell lineage relationships as direct observation of cellular division does.

The effects of lineage and clonality in T-cells are of particular interest in the context of effector cell function and differentiation in response to antigenic stimuli^7^,^16^. When performing the aforementioned analysis on a subset of genes with functional relevance to CD8+ T-cell activation, differentiation and cytotoxic function, there was once again significantly greater intraclonal transcriptional similarity (*P*<0.001, Mann–Whitney *U*-test) (Fig. 2c, Supplementary Data 3). Transcriptional similarity among the progeny of a single clone did not appear to be driven by gene expression signatures associated with pre-existing subsets of memory and effector CD8+ T cell phenotypes (Supplementary Fig. 6; Supplementary Note 3). In addition to these aggregate measurements, we compared intra- and interclonal similarity at the single gene level. The genes that showed significantly more intra- than interclonal similarity were highly enriched for gene ontology annotations relating to T-cell activation and immune cell function (*P*<0.001, modified Fisher Exact test Supplementary Data 4). Interestingly, gene expression for Granzyme B (*Gzmb*)—whose protein product plays a key role in cytotoxic T-cell-mediated target cell killing—showed one of the highest levels of clonal similarity, with strong correlations in *Gzmb* expression levels between sisters (*R*^2^=0.524, *n*=43) and cousins (*R*^2^=0.517, *n*=73) as compared with unrelated cells (*R*^2^=0.002, *n*=4,544) (Fig. 2d). At the intraclonal level for CD8+ T-cells, genes which showed stronger correlation between sister cells than between cousin cells were also enriched for gene ontology terms relating to T-cell activation and immune cell function (*P*<0.05; modified Fisher Exact test Supplementary Fig. 7, Supplementary Data 5). A similar analysis in L1210 cells, which do not require activation and are not actively differentiating, demonstrated that genes with expression levels more correlated in sister cells were instead enriched for basic biological function annotations including cell metabolism and biosynthetic processes. To our knowledge, these measurements offer the first direct comparison of inter- and intraclonal variability in activated CD8+ T-cells with *a priori* knowledge of lineage relationships and, when taken together, suggest lineage-dependent transcriptional signatures corresponding to unique functional phenotypes.

### Cell cycle-dependent transcription

Recent work has demonstrated that cell cycle-dependent transcriptional profiles in single-cell RNA-seq measurements may obfuscate underlying phenotypic relationships between cells^17^. Therefore, we sought to determine whether the observed intraclonal transcriptional similarities were primarily the result of cell cycle stage proximity or lineage relationship. When released from the device, cells derived from a single clone are inherently at similar cell cycle stages by virtue of originating from common division events. Unrelated cell pairs, however, are drawn from various lineages and have, on an average, a greater difference in cell cycle proximity. Direct observation of proliferation in the device allow us to account for this confounding effect by independently determining the time since division, an approximate measurement of cell cycle progression, for each cell analysed. Differences in time since division for unrelated cell pairs were then used as a proxy for the extent to which cell cycle stage differed for each of the transcriptional similarity measurements.

Comparison of unrelated L1210 and CD8+ T-cells demonstrated that cell pairs with smaller differences in times since division were significantly more transcriptionally similar (*P*<0.001, Mann–Whitney *U*-test, Supplementary Fig. 8). For CD8+ T-cells, this effect was even more pronounced when considering a subset of genes with cell cycle-related functional annotation (*P*<0.001, Mann–Whitney *U*-test) (Fig. 2b, Supplementary Fig. 8b). Similarly, in L1210 cells, genes which showed greater expression level similarity among related cells as compared with unrelated cells were enriched for cell cycle associated gene ontology terms (Supplementary Data 4). These results suggest that global transcriptional similarity for related L1210 and CD8+ T-cells is at least partially due to cell cycle stage proximity and not entirely governed by lineage relationships. However, this dependence on cell cycle stage was less pronounced for a subset of genes in CD8+ T-cells with functional annotations relating to T-cell activation and function (Fig. 2c, Supplementary Fig. 8c). Expression levels of these genes—including *Gzmb*—showed a strong correlation between related cells but no correlation with time since division (*P*=0.005, Fisher’s *z* transformation, Fig. 2d,e). Furthermore, when limiting the transcriptional similarity analysis to include only unrelated cell pairs that have similar cell cycle stages, sister and cousin cell pairs still demonstrate greater transcriptional similarity (Supplementary Fig. 8d–f). These results suggest that lineage relationships, and not cell cycle stage proximity, are the dominant factor accounting for the similar expression patterns of genes relating to T-cell function observed in single CD8+ T-cell clones.

To further examine cell cycle-dependent transcriptional patterns, we used partial least squares regression (PLSR) models to find the multivariate, weighted combination of genes—referred to as the latent variable—that covaried most significantly with single-cell measurements of time since division (Methods). After initial model construction with the entire gene set, we limited our analysis to subsets including the 300 genes that accounted for the most covariance with time since division for each cell type (Supplementary Fig. 9, Methods). Reconstructing the models with these reduced gene lists resulted in strong correlations between the latent variable scores and experimental measurements of time since division for both the CD8+ T-cells (*R*^2^=0.77±0.03 s.d., *n*=10 iterations ) and L1210 cells (*R*^2^=0.84±0.02 s.d., *n*=10 iterations) (Fig. 2f,g). Cross-validation of these models demonstrates that these transcriptional patterns are not artifactual signatures associated with variations amongst cells (Methods). Furthermore, random permutation analysis verifies that the results of each model were not due to over fitting (*P*<0.001, Mann–Whitney U-test) (Methods). Therefore, these transcriptional signatures are able to explain a majority of the gene expression variability associated with cell cycle progression. As such, these models could be used to predict cell cycle stage without prior knowledge of time since division for these cell types.

For each model, the optimized subsets of 300 genes were highly enriched for genes with cell cycle-related functional annotations (Supplementary Data 6). However, there were only 28 genes that were common to both subsets, indicating that cell cycle progression transcriptional signatures are different between the two cell types. This cell-type specificity may be in part due to dissimilarities in cell cycle regulation between L1210, a constitutively proliferating lymphocytic leukemia, and CD8+ T-cells, which require activation and growth factor stimulation to induce proliferation. Furthermore, these results are consistent with previous work that demonstrated distinct cell cycle signatures associated with various cell types of hematopoietic origin^18^. Altogether, these observations suggest that independent measurements of cell cycle-associated transcriptional patterns may be necessary to describe different cell types that have distinct mechanisms driving cell growth.

## Discussion

The platform presented offers the first means of directly linking single-cell transcriptional profiles with lineage history. We have demonstrated the utility of these measurements with results indicating both lineage and cell cycle-dependent gene expression profiles in two different cell types. While the system is limited to *in vitro* studies, it allows for the analysis of single-cell development and lineage progression under highly controlled, user-defined culture conditions. Such an approach enables the study of cell intrinsic developmental patterns that can serve as a benchmark for measurements of differentiation *in vivo*, where it may be difficult to deconvolve the effects of the microenvironment on cellular development. Specifically, this method may shed light on to the relative contributions of various CD8+ T-cell differentiation models, which have been difficult to reconcile with *in vivo* single-cell measurements alone^6^,^8^,^9^.

Although the trap structures implemented in this device are most effective for cell populations with a relatively uniform size distribution, they can be optimized for various average cell sizes with slight changes to the channel dimensions. Furthermore, the long-term fluidic stability and cell growth maintenance in this device make it amenable to use with actively proliferating cells with a wide range of doubling times. This flexibility in cell growth and morphology characteristics suggests that this platform will be broadly applicable to many existing model cell systems. In combination with recently developed computational approaches^17^ and high-throughput single-cell sequencing methods^19^,^20^, this multiplexing of data may allow for more sensitive analyses of single-cell RNA-seq profiles and help to distinguish subtle, but meaningful, functional signatures from cell cycle in various biological contexts.

## Methods

### Device fabrication and system setup

All devices were fabricated in 6-inch silicon-on-insulator wafers with 17-μm deep flow channels created with deep reactive ion etching and an anodically bonded Pyrex lid. Each six inch silicon wafer yields 100 devices. Fluidic connections were established by securing the devices to a Teflon manifold with PEEK tubing aligned to the access ports. This manifold was secured to a copper clamp maintained at 37 °C with a recirculating water bath (Thermo Scientific). Pressure-driven flow in the device was controlled with electronic pressure regulators (Proportion Air). All fluids were pressurized with 5% CO_2_ (Airgas) to maintain long-term pH stability of the culture medium for cell growth. Time-lapse imaging was conducted with a custom LabView program (National Instruments) which drove a TTL-triggered white LED light-source (ThorLabs) for illumination as well as two automated stages (Newport), which traversed the *x* and *y* axes to capture multiple fields of view for each frame.

### Single cell culture

Single cells were manually loaded into the device by introducing a cell sample at a concentration of 2 × 10^5^ cells per ml into the left bypass channel (Fig. 1, port with pressure P2) and flowing it into the trap lanes (P2>P3). The fluidic design of the system ensures targeted loading of single cells while avoiding capturing multiple cells in each lane (Supplementary Movie 1, Supplementary Note 1). For long-term growth and kinetics measurements, a single cell was loaded in each of the 20 lanes of the device. For the lineage RNA-seq measurements, one cell was loaded in each of the first eight lanes in order to enable imaging of all lineages during cell release.

Once a single cell was loaded in each lane, the bypass channels were flushed to remove any remaining untrapped cells. For continued nutrient repletion, cell growth media was perfused through the bypass channels at a flow rate of 100 μl h^−1^ with a pressure drop applied along the bypass channels (P1–P2 and P1–P3). A slight pressure drop was concurrently introduced across the traps (P2–P3) to ensure that the cells remained trapped and their progeny flowed downstream to unoccupied traps (Supplementary Movie 2).

### Single-cell release

After long-term proliferation, the upstream media reservoir was replaced with phosphate-buffered saline and the bypass channels were flushed. This exchange was completed within 1 min. Single cells were then retrieved from the device by briefly reversing the pressure differential across the lanes of traps (P2–P3). Once a single cell traveled to the bypass channel, the original pressure differential was reestablished and the cells once again flowed into the traps (Supplementary Movie 3). During this process, there was a constant pressure drop maintained along the bypass channels (P1–P2) that allowed each single cell that entered the bypass channel to be flushed out of the chip. The fluidic design of the system allowed for the port P2 to be maintained at atmospheric pressure, this enabled single cells to be collected directly from the tubing connected to the device (Supplementary Note 1). Furthermore, the design allowed for the release of cells from individual lanes to collect multiple single-cell lineages with each experiment (Supplementary Movie 4, Supplementary Note 1). A downstream tubing inner diameter of 75 μm resulted in a dead volume of ∼2 μl. To accommodate this dead volume, each cell was released in 5 μl of phosphate-buffered saline. For single-cell RNA-seq measurements, each cell was released directly in to a PCR tube containing 5 μl of 2 × TCL lysis buffer (Qiagen) resulting in a total volume of 10 μl of single-cell lysate. These samples were immediately frozen on dry ice and subsequently stored at −80 °C before library preparation and sequencing.

### Single cell RNA-seq

Single-cell RNA isolation, cDNA library synthesis, next-generation sequencing, read alignment and gene expression estimation were performed as described previously^15^. Briefly, Smart-Seq2 whole-transcriptome amplification and library preparation was performed on harvested single cells^21^. Single-cell libraries were then sequenced on a NextSeq500 using 30-bp paired end reads to an average depth of 1,229,637±60,907 reads (s.e.m.), and expression estimates (transcripts per million; TPM) for all UCSC-annotated mouse genes (mm10) were calculated using RNA-seq by expectation maximization (RSEM). The average transcriptomic alignment percentage was 64.3±0.63% (s.e.m.) and the average number of detected genes was 6,925±170 (s.e.m.) (Supplementary Data 7).

### Cell culture and primary cell preparation

L1210 murine lymphocytic leukemia cells (ATCC CCL219) were cultured in RPMI 1640 (Gibco) with 10% fetal bovine serum and 1% penicillin-streptomycin solution (Gibco). The L1210 cells used for single-cell RNA-seq were from cultures that had been passaged less than 15 times after the initial thaw of the ATCC aliquot. CD8+ T-cells were isolated from a mixed gender, C57BL/6J mice ranging in age from 4 to 16 weeks (six mice in total). After splenocyte isolation and red blood cell lysis with ACK buffer (Gibco), CD8+ T-cells were purified using a MACS-based CD8a T-cell isolation kit (Miltenyi Biotec). These cells were cultured in RPMI 1640 (Gibco) with 10% fetal bovine serum, 55 μM 2-mercaptoethanol (Gibco), 1% penicillin-streptomycin solution (Gibco) and 100 U ml^−1^ IL2 (PeproTech). The CD8+ T-cells were activated with 5 μg ml^−1^ plate-bound anti-mouse CD3 (clone: 145-2C11, BioLegend catalog number 100314) and 2 μg ml^−1^ of anti-mouse CD28 (clone: 37.51, BioLegend catalog number 102112) in solution for 30 h before loading in to the device.

Bulk doubling time measurements were determined by fitting an exponential proliferation model to cell count data collected at various time points with a Coulter Counter (Beckman Coulter) for cultures of L1210 and activated CD8+ T-cells.

Animals were cared for in accordance with federal, state and local guidelines following a protocol approved by the Department of Comparative Medicine at MIT.

### Image analysis

Lineage relationships and time-since-division measurements were determined by manually tracking division events and subsequent trap locations for single cells throughout time-lapse image stacks (ImageJ). To couple this information to single-cell RNA-seq measurements, the cell release process was recorded and each cell was manually assigned its corresponding sample ID.

### Gene expression data pre-processing

All analysis was performed on log-transformed expression level measurements (ln(TPM+1)). Any cell doublets (as observed during single-cell release), as well as single-cell libraries with <1,000 mapped genes, were excluded from further analysis. All genes which were expressed at a level of ln(TPM+1)>1 in >10% of cells were included in the final analysis. These constraints yielded 97 single CD8+ T-cells (out of 106 total) and 80 single L1210 cells (out of 88 total) with 9,997 and 10,658 genes for further analysis, respectively. To account for technical variations leading to different detection efficiencies for each single-cell library, we assigned observation weights to each single-cell/gene pair as described previously^4^. These weights were used for all subsequent analyses.

### Transcriptional similarity comparison

For the analysis of the full gene lists, as well as cell cycle and T-cell-specific gene lists (Supplementary Data 1,2,3), weighted Euclidean distance was used as a metric for transcriptional similarity between cell pairs. These aggregate measurements of pairwise Euclidean distances for sister cells, cousin cells and unrelated cells were compared with a Mann–Whitney *U*-test. *P* values for these tests were Bonferroni adjusted to account for multiple hypothesis testing. These analyses were robust to the use of different distance metrics, such as Spearman distance, which yielded the same results (Supplementary Fig. 4). All statistical analysis was performed in MATLAB (Mathworks).

### Partial least squares regression (PLSR) modeling

All PLSR modelling was performed with the commercially available PLS Toolbox for MATLAB (EigenVector Research). Single-latent variable models were computed with single-cell time since division measurements used to construct the response variable and corresponding single-cell gene expression measurements used as predictor variables. For variable selection, the models were iteratively constructed with 90% of the data and genes were sorted based on the average variable importance to the projection (VIP) scores across 10 iterations. To determine the optimal number of top-ranked genes to include, the model was iteratively re-run with an increasing number of genes (Supplementary Fig. 9). For the final model, we chose to use the genes with the top 300 VIP scores; at this point, the addition of more genes did not appear to increase the amount of variance captured by the cross validated model. These genes were subsequently used as predictor variables for construction of the final models. The final models were cross-validated by iteratively constructing models with 90% of cells and using them to find the latent variable scores for the remaining 10%. The mean coefficients of determination (± s.d.) across all 10 iterations are reported in Fig. 2e,f. The final models were tested for overfitting by randomly permuting the response variables, re-calculating the model and determining the permuted model residuals. A Mann–Whitney *U*-test demonstrated significantly (*P*<0.001) smaller residuals for the original models as compared with the permuted models, indicating that the model is not overfit for either cell type.

### Gene-annotation enrichment analysis

Functional enrichment analysis was performed with DAVID v6.7 ref. 22. The gene lists used for inter- and intraclonal transcription comparison (Supplementary Data 4) include genes that demonstrated significantly greater intra- than interclonal expression similarity (false discovery rate (FDR)<0.05). The lists used to analyse genes which showed greater transcriptional similarity between sister cells than between cousin cells (Supplementary Data 5) include genes with the top 1% of *ρ*_diff_ values for both cell types (Supplementary Fig. 7). The lists used to analyse cell cycle stage-dependent transcription (Supplementary Data 6) include genes with the top 300 VIP scores as determined by PLSR modelling for the full gene lists of L1210 and CD8+ T-cells. The full lists of detected genes in each cell type were used as the background gene lists for all enrichment measurements.

## 

## Additional information

**Accession codes:** The RNA-seq data generated in this study has been deposited into the Gene Expression Omnibus database hosted at the National Center for Biotechnology Information under the accession code GSE74923.

**How to cite this article:** Kimmerling, R. J. *et al*. A microfluidic platform enabling single cell RNA-seq of multigenerational lineages. *Nat. Commun.* 7:10220 doi: 10.1038/ncomms10220 (2016).

## Supplementary Material

Supplementary Information Supplementary Figures 1-9, Supplementary Notes 1-3 and Supplementary References

Supplementary Data 1 Complete gene lists for L1210 (10,658 genes) and CD8+ T cells (9,997 genes) after filtering for genes that are expressed at a level of ln(TPM+1)>1 for at least 10% of cells.

Supplementary Data 2 Subset of genes with cell cycle-related gene annotation used for comparison of Euclidean distances between cell pairs for CD8+ T cells (688 genes). The list was compiled as described in Buettner et al.

Supplementary Data 3 List of gene ontology terms used to compile a subset of genes relating to T cell activation and function as well as the corresponding gene list (142 genes). This list was used for comparison of Euclidean distances between CD8+ T cell pairs.

Supplementary Data 4 Full gene list for CD8+ T cells and L1210 cells ranked by false discovery rate (FDR) values for Mann-Whitney U tests comparing expression level differences between related cell pairs to expression level differences between non-related cell pairs. The lowest FDR values indicate genes that show more transcriptional similarity between related cells than between unrelated cells. Genes with FDR values falling below 0.05 (852 and 653 genes for CD8+ and L1210, respectively) are highlighted in red. Gene ontology terms that are enriched in these gene subsets are listed with their corresponding p values (highlighted in blue).

Supplementary Data 5 Genes with ?diff values greater than the top 1% threshold defined by a null distribution for CD8+ T cells and L1210 cells (Supplementary Fig. 5) are listed and highlighted in red. The gene ontology terms that are enriched in these gene subsets are listed with their corresponding p values (highlighted in blue).

Supplementary Data 6 Full gene lists for L1210 and CD8+ T cells ranked by average VIP scores determined from 10 iterations of partial least squares regression (PLSR) modeling with 90% of the single-cell time since division measurements used as the response variable for each iteration. The gene subsets used for the final PLSR model - which include the genes with the top 300 VIP scores for each cell type - are highlighted in red. The gene ontology terms that are enriched in each of these 300 gene subsets are listed with their corresponding p values (highlighted in blue).

Supplementary Data 7 Quality metrics for each single-cell RNA-seq sample.

Supplementary Movie 1 Demonstration of the process used to load a single cell per lane in the hydrodynamic trap array (2X playback speed). For details regarding the fluidic operation involved in this process, see Supplementary Note 1.

Supplementary Movie 2 Time-lapse imaging of a single L1210 cell lineage for 36 hours in a lane of the hydrodynamic trap array

Supplementary Movie 3 Demonstration of the fluidic process used for releasing single cells from the device. Four L1210 cells are individually released into the bypass channel of the hydrodynamic trap array.

Supplementary Movie 4 COMSOL simulation of a subset of the hydrodynamic trap array demonstrating differences in the pressure at which the flow changes direction in each lane. The arrows in the animation indicate flow direction and magnitude while the color overlay indicates local pressure within the channel. Throughout the simulation, the value of the output pressure (P3, bottom right) is gradually increased while all other pressures (P1, P2) are held constant.
