# Supplementary material for: A microfluidic platform enabling single-cell RNA-seq of multigenerational lineages
Source: Nat Commun. 2016 Jan 6;7:10220. doi: 10.1038/ncomms10220 (PMC4729820; doi:10.1038/ncomms10220)
Supplement: Supplementary Information — Supplementary Figures 1-9, Supplementary Notes 1-3 and Supplementary References [file ncomms10220-s1.pdf]

## Supplementary Figures

### Supplementary Figure 1

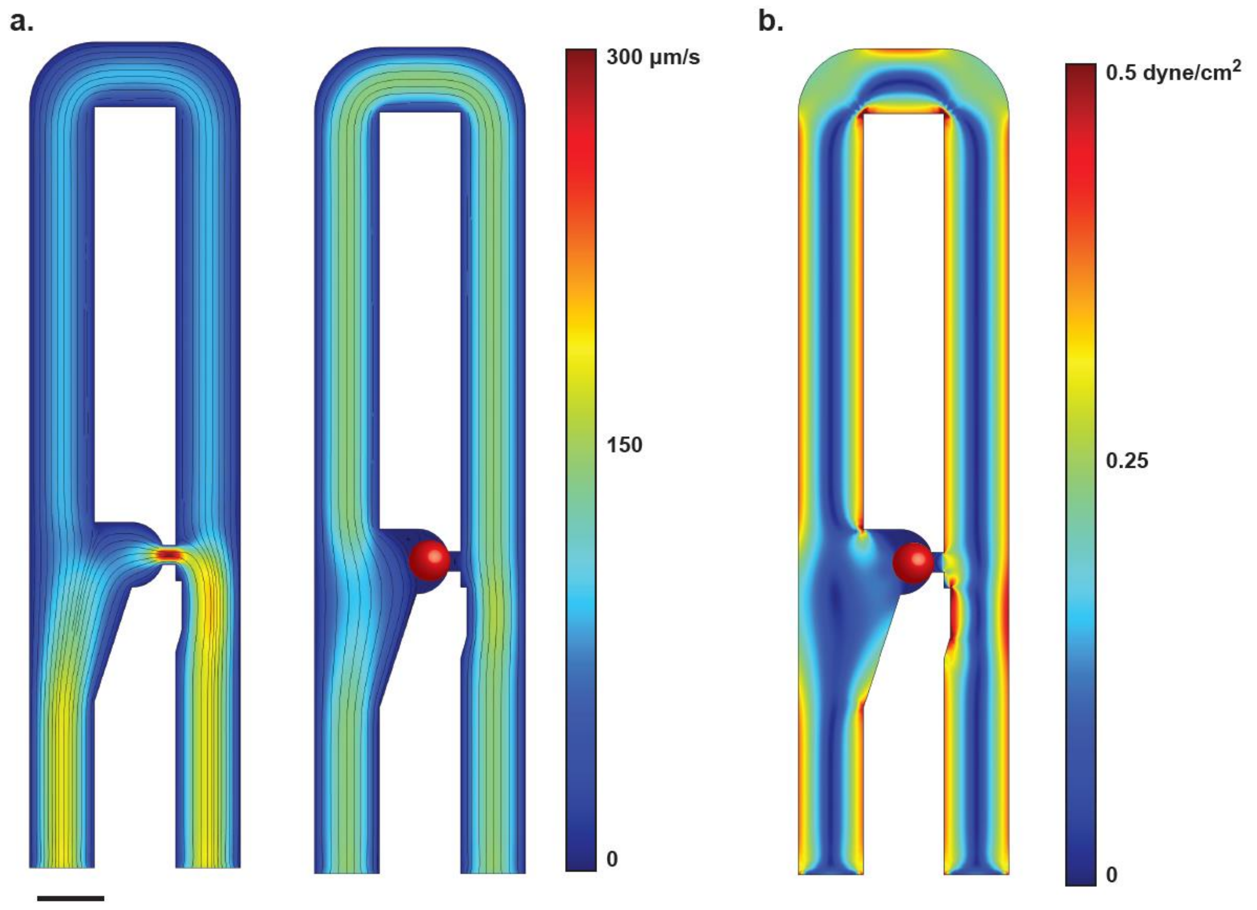


**Supplementary Figure 1** | COMSOL simulation demonstrating flow characteristics in hydrodynamic trap structures. **(a)** Flow field within the hydrodynamic traps before (left) and after (right) single-cell capture. **(b)** Shear stresses within the flow field and along the surface of a trapped cell. All simulations were calculated based on a mean flow velocity of 100 µm/s, the approximate velocity observed during experimental operation. Scale bar represents 20 microns.

## Supplementary Figure 2

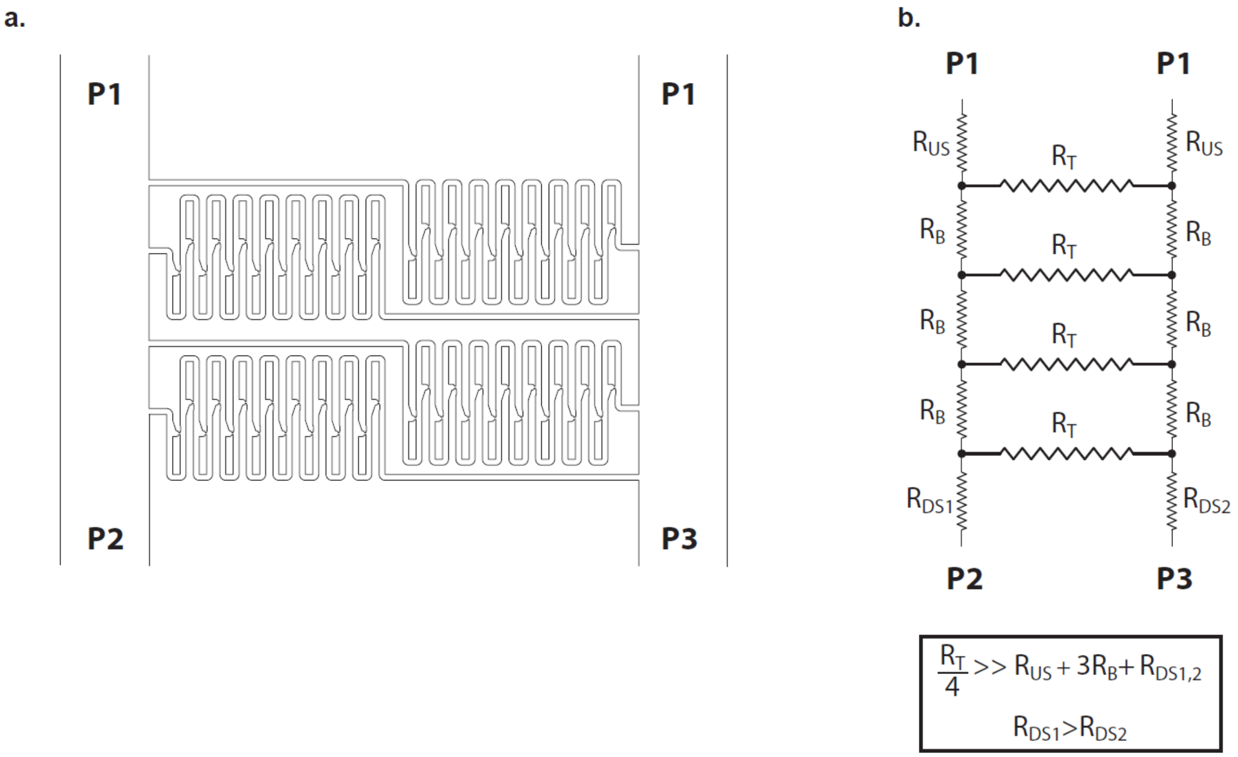


**Supplementary Figure 2 |** Schematic showing **(a)** a reduced subset of the hydrodynamic trap array demonstrating the arrangement of individually addressable trap lanes and **(b)** a resistor diagram representing the fluidic resistances corresponding to the reduced array schematic. The device performance relies on the fluidic resistance across each trap lane ($R_T$) being significantly higher than the resistance along each bypass channel ($R_B$). For a full description of how the fluidic design enables loading and releasing cells from multiple lanes see **Supplementary Note 1.** For a demonstration of loading single cells in to multiple lanes see **Supplementary Movie 1.**

**Supplementary Figure 3**

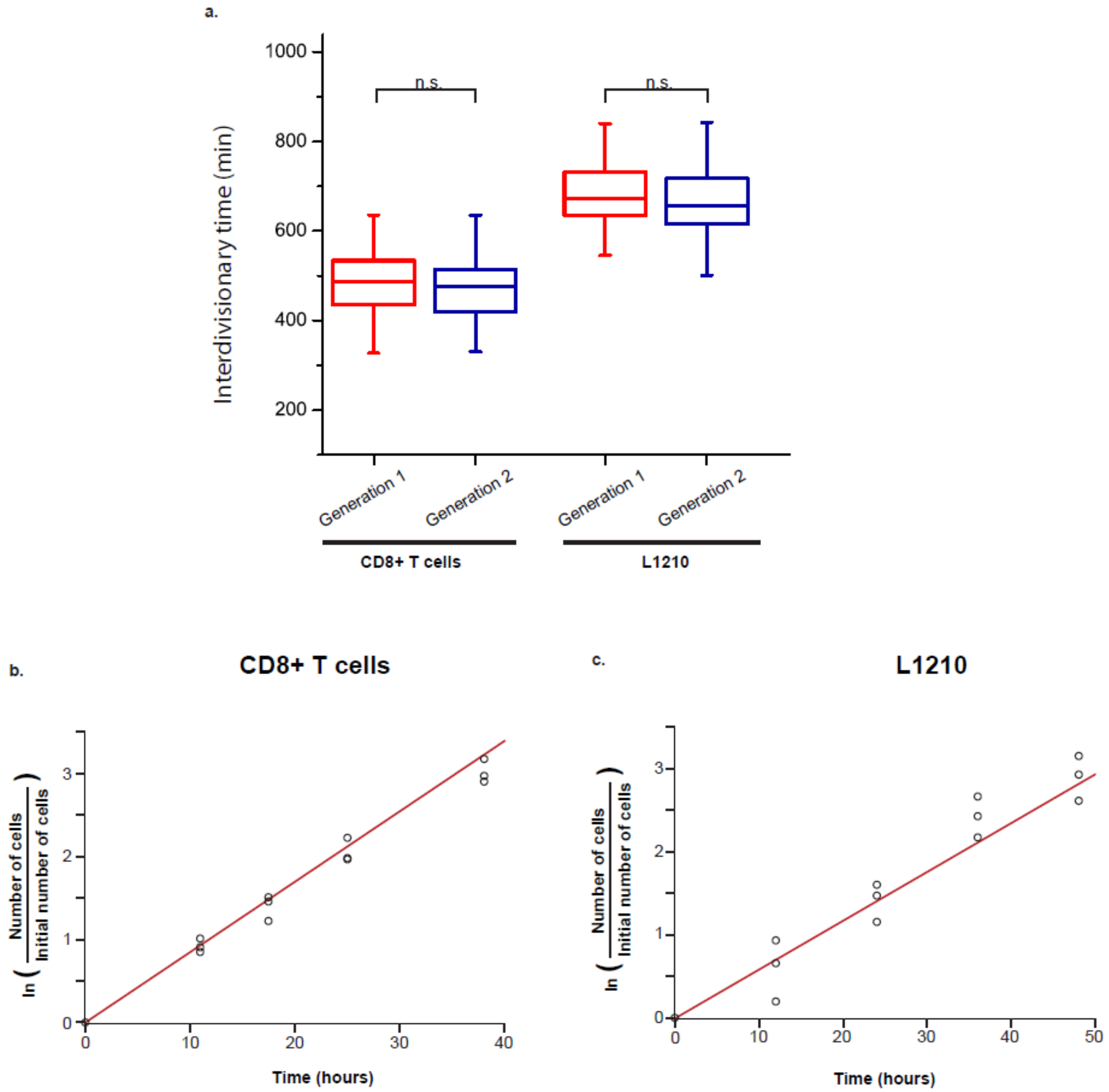


**Supplementary Figure 3 |** Cell growth stability in the device. **(a)** Comparison of single-cell interdivisionary time measurements for the first and second generations of division collected for CD8+ T cells (left, n = 168 and 131 for generations 1 and 2 respectively) and L1210 cells (right, n = 48 and 92 for generations 1 and 2 respectively) in the device. The groups were compared with a Mann-Whitney U test. Not-significant (n.s.) indicates a p-value greater than 0.05. **(b)** Proliferation measurements of CD8+ T cells grown in bulk culture collected by periodically counting cell concentration with a Coulter Counter. Points represent triplicate experiments normalized to the initial number of cells for each replicate. The red line represents the expected growth kinetics based on the mean interdivisionary time collected in the trap array (492 minutes) **(c)** Same analysis as **(b)** for L1210 cells which had a mean interdivisionary time of 698 minutes in the trap array.

**Supplementary Figure 4**

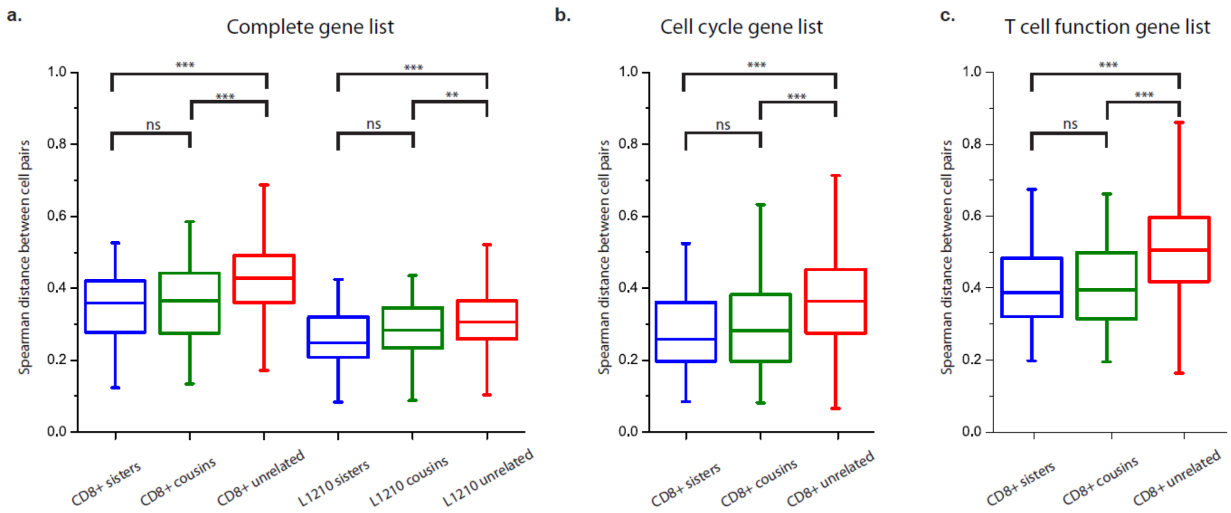


**Supplementary Figure 4 |** Comparison of Spearman distances between sister cells, cousin cells, and unrelated cells for CD8+ T cells (*n* = 43, 73 and 4,544 respectively) and L1210 cells (*n* = 37, 60 and 3,064 respectively) as in **Figure 2** for **(a)** the entire gene sets for both cell types (9,997 genes and 10,658 genes for CD8+ T cells and L1210, respectively) **(b)** a subset of genes with cell cycle related gene annotations (688 genes total, **Supplementary Table 2**) for CD8+ T cells and **(c)** a subset of genes with gene annotations related to T cell activation and function (142 genes total, **Supplementary Table 3**) for CD8+ T cells. Because Spearman distance is a rank-based metric, the observation weights determined for each cell/gene pair did not apply. Instead, the analysis was limited to genes with a mean expression level of ln(TPM+1) greater than 3 in order to reduce the noise associated with rank-ordering low-expression-level genes. Groups were compared with a Mann-Whitney U test. After Bonferroni correction: * p<0.05, ** p<0.01, ***p<0.001.

## Supplementary Figure 5

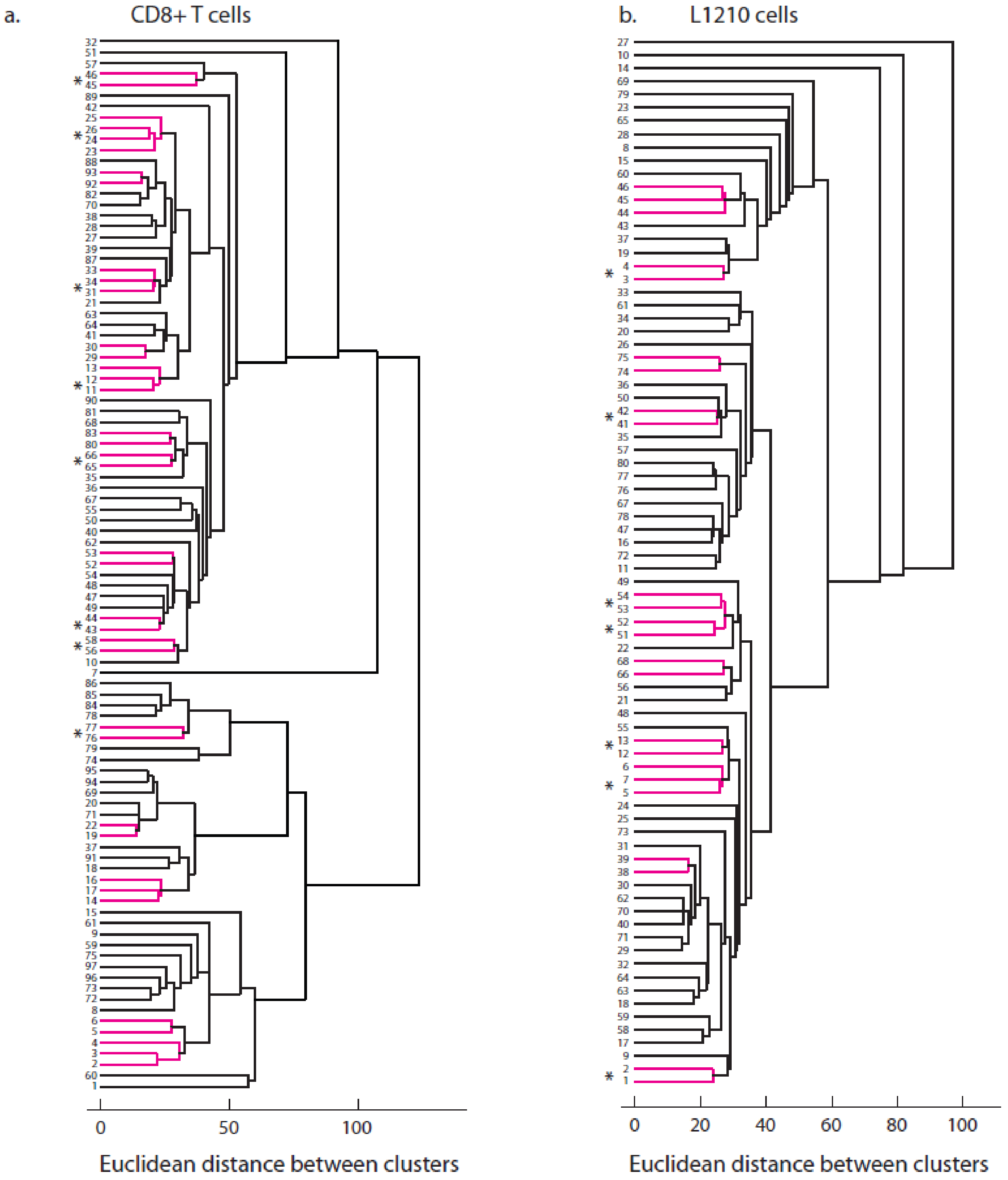


**Supplementary Figure 5 |** Unsupervised clustering dendrograms for **(a)** CD8+ T cells and **(b)** L1210 cells. Branches highlighted in magenta indicate cells that clustered most closely with another cell known to be of the same lineage while asterisks indicate cells that were clustered most closely with the cell known to be their sister. These cases indicate successful reconstruction of lineage relationships with unsupervised clustering. For methods and interpretation see **Supplementary Note 2**.

## Supplementary Figure 6

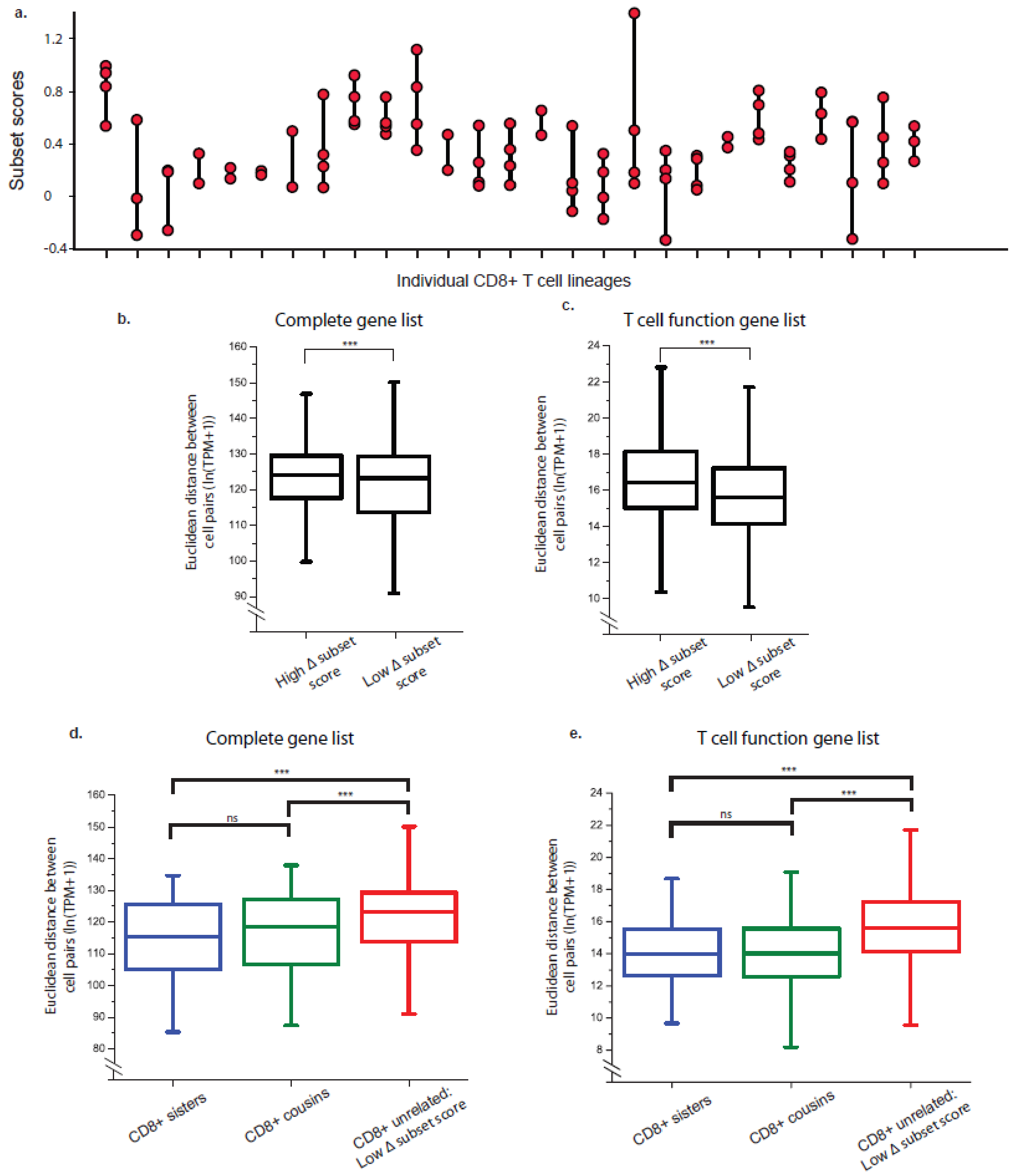


**Supplementary Figure 6 |** CD8+ T cell subset scoring. **(a)** Plot of gene expression scores indicating whether each CD8+ T cell displayed a transcriptional profile consistent with an activated-memory or activated-naïve cell. For description of how subset scores were assigned, see **Supplementary Note 3.**

To determine the effect of pre-existing CD8+ T cell subsets on transcriptional similarity between cells, unrelated pairs of CD8+ T cells were ranked by the absolute value of the difference in their subset scores (Δ subset score) and the dataset was split in thirds to produce groups with high (top third, n = 1,514) and low (bottom third, n = 1,514) differences in

subset scores. The Euclidean distances between cell pairs in each of these groups was then compared with a Mann-Whitney U test for **(b)** the entire gene list (9,997 genes) and **(c)** a subset of genes relating to T cell activation and function (142 genes, **Supplementary Table 3**). Both of these comparisons revealed that cells with a smaller difference in subset scores were more transcriptionally similar, with the effect appearing to be more pronounced in the subset of genes relating to T cell function.

To reduce the effects of pre-existing CD8+ T cell subsets when comparing transcriptional similarity, Euclidean distance measurements of CD8+ sister (n = 43) and cousin (n = 73) cell pairs were compared to unrelated cell pairs with the smallest differences in subset scores (low Δ subset score, n = 1,514) for **(d)** the complete gene list (9,997 genes) and **(e)** for a subset of genes relating to T cell activation and function (142 genes**, Supplementary Table 3**). These results indicate that unrelated cell pairs with similar subset scores still show less transcriptional similarity than related cell pairs which suggests that, although pre-existing CD8+ T cell subsets may partially contribute to inter-lineage transcriptional variability, they are not the main drivers of differences in transcriptional similarity between related and unrelated cell pairs. After Bonferroni correction: * $p<0.05$, ** $p<0.01$, ***$p<0.001$.

## Supplementary Figure 7

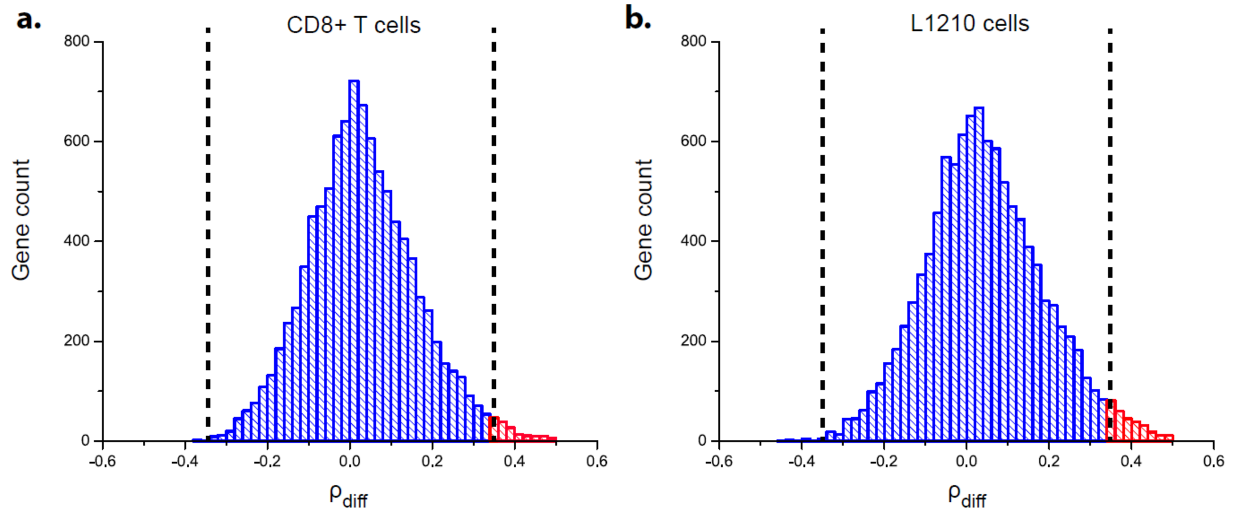


**Supplementary Figure 7 |** Comparing gene expression similarity in sister cells versus cousin cells. The difference in Spearman correlation coefficients for expression levels in sister cell pairs and cousin cell pairs ($\rho_{diff} = \rho_{sisters} - \rho_{cousins}$) was determined for each gene in **(a)** CD8+ T cells (9,997 genes total) and **(b)** L1210 cells (10,658 genes total). To determine genes that were expressed more similarly in sister or cousin cells, we defined an expected null distribution of $\rho_{diff}$ with a mean of zero and a standard deviation approximated as the average standard deviations of $\rho_{diff}$ we observed in these two cell types (0.15). We then defined the values of $\rho_{diff}$ corresponding to the top and bottom 1% of this distribution (± 0.349, vertical dashed lines) as the thresholds for the highest and lowest values of $\rho_{diff}$ to determine the genes that are more similarly expressed in sister cells or cousin cells, respectively. Both cell types demonstrated subsets of genes which were more similarly expressed in sisters as compared to cousins (149 genes and 288 genes for CD8+ and L1210 cells respectively, **Supplementary Table 5**). To determine the biological function of these genes, these lists were used for functional enrichment analysis for both cell types (**Supplementary Table 5**). Both cell types showed very few genes (<10) that were more similarly expressed in cousins as compared to sisters and these genes did not reveal any functional enrichment. This result is consistent with the positive skew observed for the $\rho_{diff}$ distributions for both L1210 and CD8+ T cells ($\gamma$ = 0.294 and 0.318, respectively), which suggests that, for both cell types, there are more genes with a higher correlation between sister cell expression levels than between cousin cell expression levels.

## Supplementary Figure 8

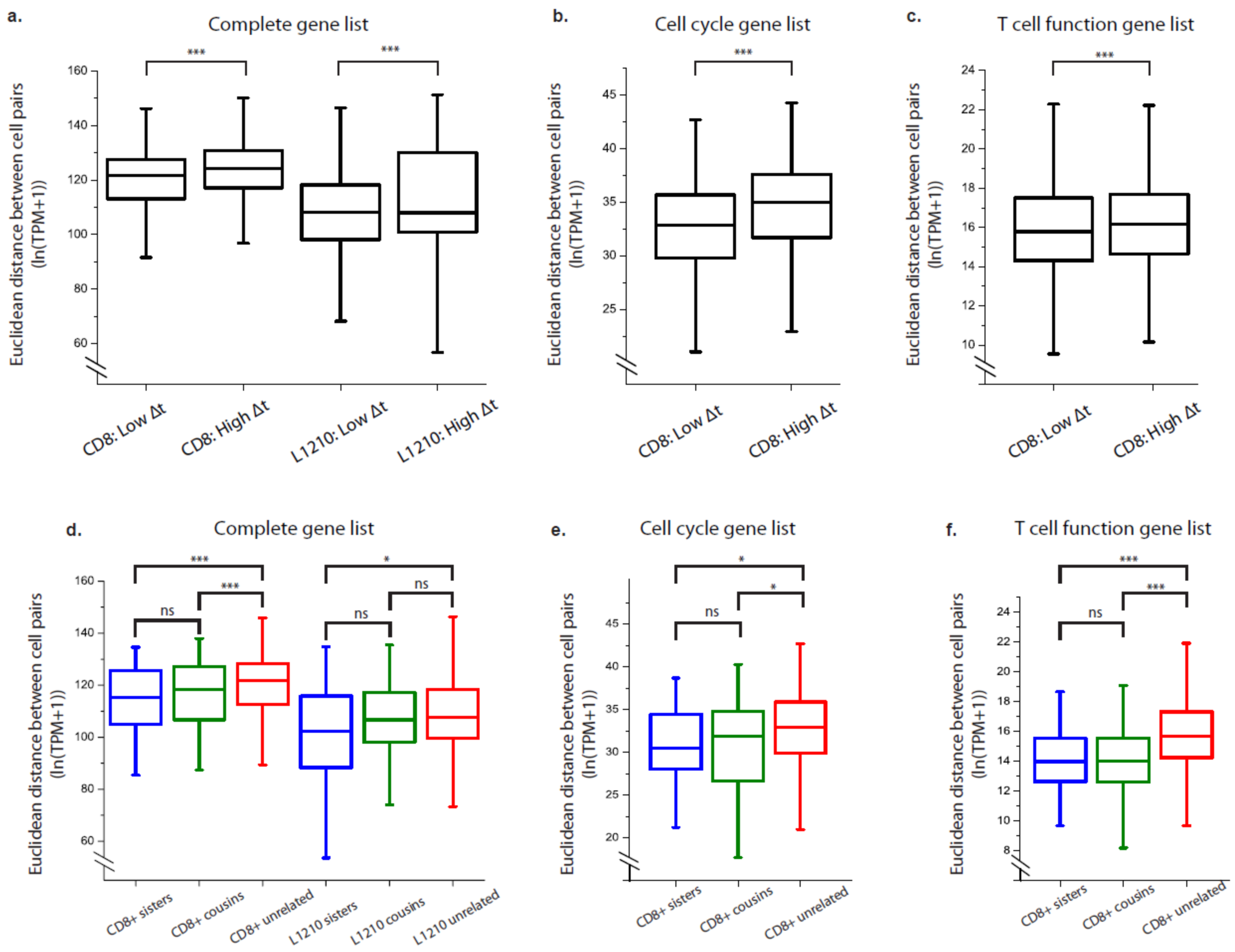


**Supplementary Figure 8 |** Comparison of transcriptional similarity between unrelated cells with differing cell cycle stage proximities. **(a)** Global gene expression-based Euclidean distances between unrelated pairs of L1210 and CD8+ T cells were ranked by their corresponding difference in times since division (Δt) and the dataset was split in thirds to produce groups with low (bottom third, n = 1,514 and 1,021 for CD8+ and L1210 cells, respectively) and high (top third, n= 1,514 and 1,021 for CD8+ and L1210 cells, respectively) Δt values corresponding to cells with more and less similar cell cycle stages, respectively. These groups were then compared with a Mann-Whitney U test. These results demonstrate that cell pairs with smaller differences in times since division are more transcriptionally similar. **(b)** Same analysis as in **(a)** applied to a subset of genes with cell cycle related gene annotations (688 genes total, **Supplementary Table 2**) for CD8+ T cells. **(c)** Same analysis as in **(a,b)** applied to a subset of genes with gene annotations related to T cell activation and function (142 genes total, **Supplementary Table 3**) for CD8+ T cells. These comparisons show that the effect of cell cycle stage proximity on transcriptional similarity is less pronounced for genes related to T cell function than for cell cycle related genes in CD8+ T cells.

To reduce the effects of cell cycle stage differences in transcriptional similarity measurements, Euclidean distance measurements of sister (n = 43 and 37 for CD8+ and L1210 respectively) and cousin cell pairs (n = 73 and 60 for CD8+ and L1210 respectively) were compared to unrelated cell pairs that had a difference in times since division (Δt) of less than 2 hours (n = 1,006 and 495 for CD8+ and L1210 respectively) for **(d)** the entire gene list (9,997 genes and 10,658 genes for CD8+ T cells and L1210 respectively). **(e)** Same analysis as in **(d)** for genes relating to cell cycle progression in CD8+ T cells (688 genes total, **Supplementary Table 2**). **(f)** Same analysis as in **(e)** for genes relating to T cell activation

and function (142 genes total, **Supplementary Table 3**). These results indicate that unrelated cell pairs with similar cell cycle stages (small $\Delta t$) still show less transcriptional similarity than related cells, which suggests that cell cycle proximity is not the main driver of differences in transcriptional similarity between related and unrelated cell pairs. After Bonferroni correction: * $p<0.05$, ** $p<0.01$, *** $p<0.001$.

## Supplementary Figure 9

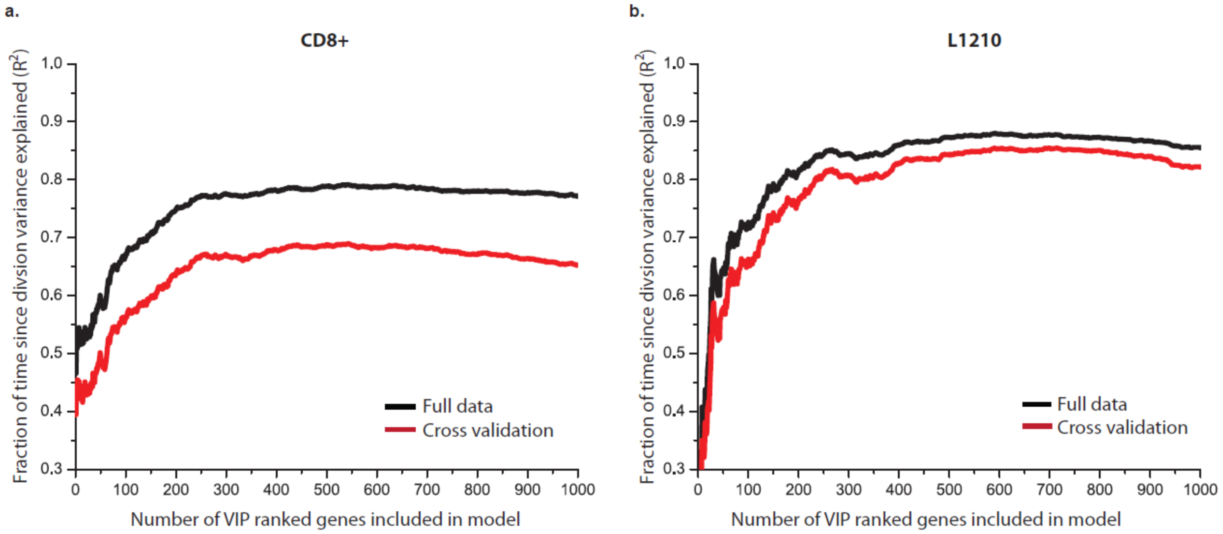


**Supplementary Figure 9 |** Plot of the coefficients of determination ($R^2$) between the first latent variable scores and single-cell measurements of time since division as a function of the number of VIP ranked genes included in the model for **(a)** CD8+ T cells and **(b)** L1210 cells. These $R^2$ values indicate the fraction of gene expression variance due to time since division that is explained by these models. The variance explained by the full model (black) and cross-validated model (red) are both included for comparison. The amount of variance explained by the model appears to plateau at around 300 genes for each cell type. For this reason, we used a subset of genes with the top 300 VIP scores for the final model construction presented in **Figure 2 e, f.**

## Supplementary Notes

**Supplementary Note 1 |** Device operation.

**Loading cells into multiple lanes.** When loading single cells into the trap array, a plug of cells at a concentration of $2x10^5$ is first loaded into the left bypass channel by loading a sample of cells in the vial for the port labeled P2 and pressurizing the system such that P2>P1 and P2=P3 **(Supplementary Fig. 2)**. Once cells are in the left bypass channel of the system, the pressures are adjusted such that P2>P1 and P3=P1. Seeing as the total fluidic resistance along the bypass channels of the device (in the resistor schematic: $R_{us}$, 3 $R_B$, and $R_{ds}$ connected in series) is significantly lower than the total resistance across the lanes of traps (in the resistor schematic: 4 $R_T$ connected in parallel), for a fixed pressure differential ($\Delta P$ = P2-P1 = P2-P3), a larger fraction of flow will be directed along the bypass channel as compared with the flow directed across the trap lanes. For this reason, a single cell can be brought in close proximity to a trap lane entrance and the pressure P1 can periodically be toggled to be higher than P2 to allow the cell to slowly drift in to the trap lane without traveling further down the bypass channel (**Supplementary Movie 1**). This design allows for the precise loading of a single cell in to each lane of traps without capturing multiple cells in each lane.

**Single cell release**. The downstream resistance leading to port P2 ($R_{DS1}$) is higher than the downstream resistance leading to port P3 ($R_{DS2}$) due to a longer length of tubing connecting port P2 to its corresponding pressure reservoir. For this reason, a fixed pressure drop (P1-P2 = P1-P3) will generate flow in the direction of cell trapping (left to right in **Supplementary Fig. 2a**) even while P2 and P3 are maintained at the same pressure. This allows the pressure reservoir for P2 to be opened and the corresponding connection tube left open to atmospheric pressure. To release cells, P3 is increased until the flow direction changes direction and, as soon as a cell is released in to the bypass channel, the tubing leading to port P2 on the device is placed in a PCR tube to collect the volume flushed from the system along with the single cell it contains.

**Releasing cells from multiple lanes.** For releasing cells from each lane of traps, the pressures on the system are adjusted such that P1≫P2 while P2 and P3 are both set to atmospheric pressure. This pressure balance ensures that fluid is constantly flowing through the bypass channels with slight flow directed across the trap lanes to keep the cells captured. Once buffer is flowing through the bypass channels, the pressure P3 is periodically increased such that the flow direction is reversed and cells begin to exit the trap lanes (**Supplementary Movie 3**). Each lane of traps is located at a slightly different vertical position along the bypass channels and therefore each lane has a unique value of P3 at which flow reverses direction and cells begin to exit the traps (**Supplementary Movie 4**). Flow originally reverses direction in the lower left-most trap lane and, as P3 is increased, propagates up the array with the top right-most lane reversing direction last. This variability in the pressure required to release cells from each lane, coupled with the difference in proximity to the bypass channels of the left and right columns of lanes, allows for multiple lanes of cells to be released independently**.**

**Supplementary Note 2 |** Unsupervised clustering and lineage reconstruction.

To perform unsupervised clustering of single CD8+ T cells and L1210 cells, we first constructed lists of highly variable genes using the Seurat package in R (Jackstraw method)[2]. This resulted in subsets of 777 and 647 highly variable genes for CD8+ T cells and L1210 cells, respectively. We then performed agglomerative clustering for each cell type in MATLAB using Ward’s method after mean centering and unit variance adjustment (**Supplementary Figure 5**). We compared the results of hierarchical clustering for these two cells types to the known lineage information gathered with time-lapse microscopy. For the CD8+ T cells, 16 out of 97 cells were correctly paired with their sister cell, while 38 out of 97 cells were paired with a cell from the same lineage (i.e. cousin or sister cell). The probabilities of these sister and lineage pairs occurring by random chance, as modeled with a binomial distribution, are $5.5x10^{-15}$ and $8.9x10^{-32}$, respectively. Similarly, for the L1210 cells, 14 out of 80 cells were correctly paired with their sister cell, while 24 out of 80 cells were paired with a cell from the correct lineage. In this case, the probabilities of these sister and lineage pairs occurring by random chance, once again as modeled with a binomial distribution, are $1.5x10^{-12}$ and $1.1x10^{-15}$, respectively.

These results suggest that unsupervised clustering of single cell transcriptional profiles can effectively reconstruct lineage membership although with a much lower accuracy compared to lineage tracking by means of direct observation of cell division via time-lapse microscopy. Furthermore, it is important to note that in other single cell experimental designs in which cells are isolated using methods including FACS sorting, the Fluidigm C1 platform, or micromanipulation, there will likely be either larger sample sizes or fewer closely related cells, which will reduce the efficacy of computationally reconstructing lineage relationships. This suggests that direct observation of single cell lineage upstream of transcriptional profiles offer a higher degree of accuracy in analyzing lineage dependent transcriptional signatures.

**Supplementary Note 3 |** CD8+ T cell subset scoring**.**

In previously published work, Hinrichs et al. sorted naïve and memory CD8+ T cells from murine splenocytes prior to activation with anti-CD3 and anti-CD28 – the same activation scheme used in this study[3]. Microarray analysis of these two populations revealed sets of genes that were differentially regulated in naïve cells after activation (activated-naïve cells) relative to memory cells after activation (activated-memory cells; MSigDB systematic names M3660 and M3662 for up-regulated and down-regulated gene lists, respectively). To determine a subset score for each cell, we calculated the weighted mean of expression levels for up-regulated genes and subtracted the weighted mean of expression levels for down-regulated genes. Each vertical line in **Supplementary Figure 6** indicates a single CD8+ T cell lineage with points indicating individual cells. The subset scores do not appear to demonstrate lineage-dependent values that partition into clear groups of high and low scores. This does not fully exclude the effects of pre-existing CD8+ T cell subsets but suggests that the lineages studied here do not display diverging transcriptional profiles driven primarily by the phenotype of the founding cell.

## Supplementary References


1. Buettner, F. et al. Computational analysis of cell-to-cell heterogeneity in single-cell RNA-sequencing data reveals hidden subpopulations of cells. *Nature Biotechnology* **33**, 155-160 (2015).
2. Satija, R., Farrell, J.A., Gennert, D., Schier, A.F. & Regev, A. Spatial reconstruction of single-cell gene expression data. *Nature Biotechnology* **33**, 495-U206 (2015).
3. Hinrichs, C.S. et al. Adoptively transferred effector cells derived from naive rather than central memory CD8(+) T cells mediate superior antitumor immunity. *Proceedings of the National Academy of Sciences of the United States of America* **106**, 17469-17474 (2009).
